# Adaptive Foraging in Dynamic Environments Using Scale-Free Interaction Networks

**DOI:** 10.3389/frobt.2020.00086

**Published:** 2020-07-09

**Authors:** Ilja Rausch, Pieter Simoens, Yara Khaluf

**Affiliations:** IDLab - Department of Information Technology, Ghent University—IMEC, Ghent, Belgium

**Keywords:** swarm robotics, foraging, collective decision-making, scale-free networks, dynamic environments, adaptive swarm

## Abstract

Group interactions are widely observed in nature to optimize a set of critical collective behaviors, most notably sensing and decision making in uncertain environments. Nevertheless, these interactions are commonly modeled using local (proximity) networks, in which individuals interact within a certain spatial range. Recently, other interaction topologies have been revealed to support the emergence of higher levels of scalability and rapid information exchange. One prominent example is scale-free networks. In this study, we aim to examine the impact of scale-free communication when implemented for a swarm foraging task in dynamic environments. We model dynamic (uncertain) environments in terms of changes in food density and analyze the collective response of a simulated swarm with communication topology given by either proximity or scale-free networks. Our results suggest that scale-free networks accelerate the process of building up a rapid collective response to cope with the environment changes. However, this comes at the cost of lower coherence of the collective decision. Moreover, our findings suggest that the use of scale-free networks can improve swarm performance due to two side-effects introduced by using long-range interactions and frequent network regeneration. The former is a topological consequence, while the latter is a necessity due to robot motion. These two effects lead to reduced spatial correlations of a robot's behavior with its neighborhood and to an enhanced opinion mixing, i.e., more diversified information sampling. These insights were obtained by comparing the swarm performance in presence of scale-free networks to scenarios with alternative network topologies, and proximity networks with and without packet loss.

## 1. Introduction

The efficiency of the information sharing mechanisms used by individuals during group decision processes determines to a large extent the fitness of the group decision. In nature, collective systems consist of a high number of individuals living in large and unknown environments, and needing to perform complex tasks to survive. Among the many examples of collective decision-making is choosing a new site to build their home (Richardson et al., 2018), or deciding among a number of foraging patches (Michelena et al., 2009). Despite the high diversity of tasks, uncertainty and complexity are common features. Hence, individuals apply information pooling to mitigate uncertainty and increase decision accuracy (Conradt, 2011). Achieving efficient opinion sampling depends to a large extent on the network topology that defines the interaction structure and opinion sharing of these individuals (Khaluf et al., 2018; Rausch et al., 2019b). The use of such network is fundamental for collective decision-making. It is generally exploited at two stages of the process (i) when spreading information on one or multiple stimuli that are initially perceived by a limited number of individuals that are able to trigger the collective decision process—e.g., a predator attack—; and (ii) when spreading the individuals' opinions or choices to achieve consensus (Khaluf et al., 2019a).

In artificial systems such as swarm robotics, collective decision-making is mostly designed in static environments (Bayındır, 2016), where options and their qualities are defined at the beginning and do not change over time. In these studies the focus is mainly on the design of efficient voting mechanisms that enable a high level of decision coherence within the shortest time possible (Khaluf et al., 2018). Alternatively, other studies were addressing the design of decision strategies that tackle the accuracy vs. speed trade-off (Valentini, 2017)—i.e., taking longer time to gather enough information and making more accurate decisions vs. exploiting the available information and taking the decision as soon as possible. In both cases, the speed of converging on a decision is a fundamental goal in the design of decision-making. The decision speed strongly depends on the interaction topology the individuals are part of, to spread stimuli or opinions during the decision-making process. Interactions in collective systems are frequently modeled using local (i.e., proximity) communication, where the neighborhood of an individual is defined spatially based on their interaction range, i.e., interacting with all peers within the individual's communication radius. Nevertheless, other interaction models such as scale-free networks were revealed in several real-world examples (Albert and Barabási, 2002; Holme, 2019). A comprehensive review on scale-free phenomena in a more general context can be found in Khaluf et al. (2017a). In various works, scale-free networks enable scalable, fast and efficient information transfer. For example, in Goh et al. (2001), authors showed how the betweenness centrality scales with the scale-free exponent. Other works showed how the ultrasmall diameter of the scale-free networks contributes to their efficiency in information transmission (Cohen and Havlin, 2003; Thivierge, 2014). Finally, scale-free topologies were studied in natural collective systems such as in Cavagna et al. (2010). In this work, the authors studied starlings flocks and suggest that collective response to predator's attacks may be achieved through scale-free behavioral correlations. Based on these studies, we extend the application of scale-free networks to artificial swarms in order to investigate the role these networks can play in improving a swarm's collective decision-making process.

A key aspect of scale-free networks is the presence of hubs—i.e., nodes with a comparably high connectivity degree—(Albert et al., 2000; Albert and Barabási, 2002). Hubs represent a small percentage of the network nodes, however, their high connectivity leads to a small network diameter. This facilitates efficient communication by enabling any two random nodes to share information over only few hops, resulting in fast information transfer (Cohen and Havlin, 2003). In this paper, we exploit this critical feature of scale-free networks to help collective systems to faster respond to changes in dynamic environments. In dynamic environments, conditions change over time and hence, the collective system needs to adapt its behavior within a short period of time in order to survive. We refer to this as the collective response time. In our study, this is the time required for the group to collectively change the intensity of its foraging activities as a response to a change in the availability of the food items.

Among many examples of collective tasks in natural systems, we select *foraging* (Liu et al., 2007) and perform our study using a simulated population of swarming robots. Foraging is a complex task used by many species to retrieve food to their homes, but beyond that it is a metaphor for many real-world robotics tasks such as search and rescue, retrieve materials for collective construction and others. In foraging, individuals (robots) need to continuously make a decision between staying at their base or leaving to forage for food items. A large body of literature has been dedicated to investigate foraging in artificial systems such as swarm robotics. These studies have addressed various research questions such as the foraging performance under the influence of physical robot interference (Lerman and Galstyan, 2002; Khaluf et al., 2016), the multi-foraging task (Campo and Dorigo, 2007)—i.e., the foraging for different types of items—or consensus achievement (Hoff et al., 2013; Khaluf et al., 2017b). Additionally, some studies have focused on how to optimize the task allocation in foraging using cost functions (Pini et al., 2013; Khaluf et al., 2019b). Also how to investigate simple probabilistic models that rely on the foraging success probability in achieving an efficient foraging behavior (Pinciroli et al., 2012). Other studies have gone further to investigate whether the performance of swarms in the foraging tasks bears a particular characteristic distribution (e.g., a power law) for any of its time or space features (Khaluf and Dorigo, 2016; Rausch et al., 2019a). Despite this intensive research effort, foraging of robot swarms in dynamic environments and the influence of different interaction models are still not well understood. However, these questions are paramount, given the prevalence of scale-free phenomena in real-world systems and admitting that most real environments are dynamic. Therefore, in this paper, we focus on the fundamental question of how the integration of a scale-free interaction structure may influence the collective response of simulated swarms to changes in food density within the foraging environment. We approach this question by analyzing the speed and coherence of the collective response to those changes. We begin with defining the robot (microscopic) and the swarm (macroscopic) behaviors in sections 2.1, 2.3, respectively. The details on generating scale-free networks from local neighborhoods are given in section 2.2. In section 2.4, we describe the experimental setup. Thereafter, in section 3 we compare the collective response of the swarm in presence and absence of scale-free interactions. We discuss our findings that suggest that the use of scale-free interactions can be advantageous due to (i) reduced correlations between a robot's decisions and those of its spatial neighbors and (ii) enhanced information spread through long-range interactions and frequent rewiring of communication links. These insights are obtained by comparing the influence of scale-free networks to scenarios with alternative random networks as well as scenarios that include packet loss. Conclusions are drawn in section 4.

## 2. Methods

### 2.1. Robot Behavior

Robots are placed in an arena that is divided into two areas: the nest and the foraging environment. Inspired by the behavior observed in harvester ants *Pogonomyrmex barbatus* (Schafer et al., 2006; Pinter-Wollman et al., 2013), each robot can switch between two essential states: *resting* and *foraging*. In the foraging state, the robot attempts to find a food item inside the foraging environment by performing a pseudo-random walk. In particular, the robot moves on a straight line until it encounters another robot or an obstacle (e.g., a wall), in which case a *collision avoidance* maneuver is initiated. By executing this maneuver, the robot attempts to move in the direction of least physical interference, as sensed by its proximity sensors. After executing the collision avoidance maneuver, the robot goes back to its standard motion following a straight line. When the robot encounters a food item, it collects this item and retrieves it back to the nest where the robot rests for a given period of time θ_*r*_.

In the resting state, the robot remains inside the nest, which is the only area where communication with other robots can take place. This is inspired by several natural systems, in which the communication occurs mainly inside the nest or the hive (Liu et al., 2007; Seeley et al., 2012; Reina et al., 2015; Valentini et al., 2016). This approach accommodates two relevant properties of foraging systems: (i) it is common that the foraging environment is significantly larger than the nest area, and hence, individual encountering rates outside the nest are negligibly low. (ii) Due to the high density of individuals inside the nest there is a high likelihood of interaction between individuals that have explored different parts of the foraging environment, and hence a more diversified sample of information about the environment can be collected.

Robots can communicate only with neighbors that are within a direct line of sight, sharing their individual experiences. This is a continuous process—i.e., each robot broadcasts at every time step its previous experience (success or failure in finding a food item) until it switches again to the foraging state. Continuous communication activity is a required choice of the experiment design to research the role of network topology in the emergent behavior (Rausch et al., 2019a).

All robots, in our study, are identical and each robot is a probabilistic finite state machine. In particular, a robot's behavior is shaped by two switching probabilities that describe at every time step the robot's likelihood to switch from foraging to resting (*P*_*f*→*r*_) or the opposite (*P*_*r*→*f*_). These probabilities are updated differently at the robot's resting and foraging states. At the foraging state, the switching probabilities are updated using the robot's foraging experience. The impact of this experience on the robot's decision-making is given by the set of two individual cues $\left\{i_{f},i_{r}\right\}\in {\mathbb{R}}_{0}^{+}\times {\mathbb{R}}_{0}^{+}$. More specifically, the cue *i*_*f*_ defines a numerical value by which the probability to switch from resting to foraging (*P*_*r*→*f*_) is increased when the robot has experienced foraging success—i.e., a discovered food item during the latest foraging attempt. The same value is used to decrease this switching probability in case of a failed foraging attempt, i.e., when the robot has spent a specific time (θ_*f*_) foraging without finding a food item. The cue *i*_*r*_ updates the robot's switching probability from foraging to resting (*P*_*f*→*r*_) in a manner that is inverse to *i*_*f*_. Besides updating the switching probabilities at the foraging state, the robot updates those while resting. This update is performed using the experience received from the robot's neighbors and is numerically given by two social cues $\left\{s_{f},s_{r}\right\}\in {\mathbb{R}}_{0}^{+}\times {\mathbb{R}}_{0}^{+}$. The social cue *s*_*f*_ is used to update the switching probability from resting to foraging (i.e., *P*_*r*→*f*_) by increasing (decreasing) *P*_*r*→*f*_ when the robot's neighbors report primarily on successful (failed) foraging attempts. Whereas, *s*_*r*_ is used to update the switching probability from foraging to resting (i.e., *P*_*f*→*r*_), inversely to *s*_*f*_. In the following we define how the switching probabilities are updated at every simulation step (as described in Rausch et al., 2019a; to prevent divergence, both probabilities were truncated between zero and one):

(1) $$\begin{array}{l} P_{r\to f}\left(t+1\right)=P_{r\to f}\left(t\right)+\delta_{\eta }\left(t\right)s_{f}+\delta_{\phi }\left(t\right)i_{f} \end{array}$$

(2) $$\begin{array}{l} P_{f\to r}\left(t+1\right)=P_{f\to r}\left(t\right)-\delta_{\eta }\left(t\right)s_{r}-\delta_{\phi }\left(t\right)i_{r}, \end{array}$$

where δ_η_(*t*) is the difference between the successful and the failed foraging attempts communicated to the robot by its neighbors. Hence, it has a positive sign when there are more successful attempts than failed ones and a negative sign otherwise. Consequently, the former increases the switching probability from resting to foraging and the latter increases the switching probability from foraging to resting. δ_η_(*t*) = 0 if the robot is not resting. Additionally, the robot's individual experience during a foraging attempt that starts at *t*_*f*_ is defined as follows:

(3) $$\begin{array}{l} \delta_{\phi }\left(t\right)=\left\{ \begin{array}{cc} +1, & \text{at }t_{if}\\ 0, & \text{if }t_{f}<t\leq t_{f}+\theta_{f}\&\text{no item is found }\\ -1, & \text{if }t>t_{f}+\theta_{f}\&\text{the robot is still foraging } \end{array}\right. \end{array}$$

where *t*_*if*_ is the (unique) time step at which the robot finds an item while in foraging state. While in the foraging state, the robot may find an item at any time *t*_*f*_ < *t*_*if*_ (i.e., it could also happen that *t*_*f*_ + θ_*f*_ < *t*_*if*_). After finding an item, i.e., subsequently to *t*_*if*_, the robot leaves the foraging state. If no item is found and the foraging time crosses the threshold θ_*f*_, then δ_ϕ_(*t*) = − 1. This increases *P*_*f*→*r*_(*t*) at every time step *t* > *t*_*f*_ + θ_*f*_, guaranteeing that the robot will probabilistically leave the foraging state at some *t*, even without finding an item. δ_ϕ_(*t*) = 0 outside of the foraging state.

**Figure 1:**
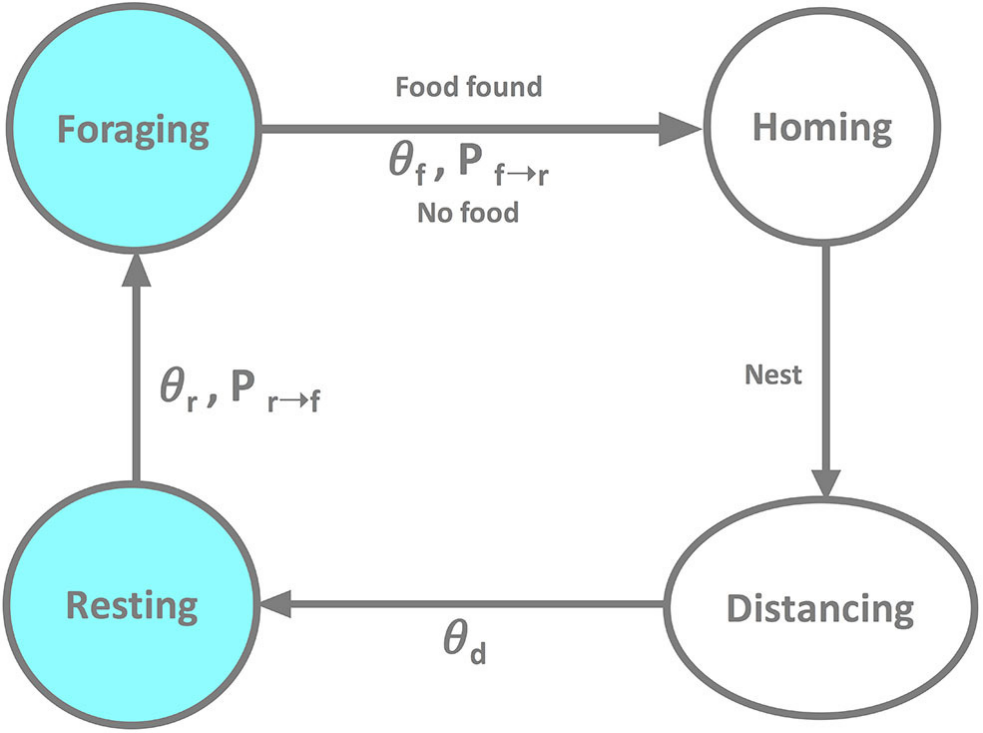
The state transition diagram of a robot performing the foraging task.

The robot behavior is illustrated in Figure 1 using a state diagram. It includes the following states: (i) foraging: after having spent at least θ_*r*_ time steps resting, the robot switches with probability *P*_*r*→*f*_ from resting to foraging. It attempts to search the foraging area for a food item to retrieve to the nest. If the robot fails to find a food item within a predefined time θ_*f*_, it switches with probability *P*_*f*→*r*_ to homing; (ii) homing: in this transitional state the robot returns to the nest, with δ_η_(*t*) = 0 and δ_ϕ_(*t*) = 0; as soon as the robot reaches nest, it switches to distancing; (iii) distancing: having returned to the nest, the robot searches for an empty spot in the nest where it can rest; similar to the homing state, distancing is a transitional state with δ_η_(*t*) = 0 and δ_ϕ_(*t*) = 0; distancing terminates after θ_*d*_ time steps and the robot switches to resting; (iv) resting: subsequent to distancing the robot rests for at least θ_*r*_ time steps after which it switches with probability *P*_*r*→*f*_ to foraging. A resting robot broadcasts “success” (or “failure”) to its neighbors if the latest foraging attempt was successful (or not), respectively. If the robot failed to leave the nest in state (i), it has no information about the foraging environment and, thus, does not broadcast any message. Throughout the entire experiment, the robot performs collision avoidance maneuvers if other robots or walls enter its proximity sensors' range (not shown in Figure 1 for better readability).

### 2.2. Robot Scale-Free Communication Network

In this section, we describe the design and implementation of the algorithm that leads to a scale-free robot communication network. An implementation of this algorithm in C++ is publicly available online^1^ (Rausch et al., 2020). The generation of a scale-free network from local neighborhoods is an iterative process, where at each time step *t* the robot communication is updated according to the following procedure:

1. Identify all connected components (*CC*s) in the resting swarm using depth-first-search. A *CC* is the maximal set of nodes (robots), where each two nodes are connected through a finite path. The *CC*s are initially derived from the spatial networks in which the robots are neighbors if they are within each other's communication radius.
2. Generate the scale-free network topology within a *CC* using preferential attachment (Albert and Barabási, 2002) as summarized in Algorithm 1. This algorithm is largely inspired by previously proposed approaches (Li and Chen, 2003; Jiang et al., 2014). We begin by selecting a sink node ν_*s*, 0_ which is the node with the highest number of neighbors within its spatial proximity—i.e., within the initial radius of *r*_*s*_ = 1.25 m. Within this *r*_*s*_, each spatial neighbor ν_*s, i*_ is linked to ν_*s*, 0_, creating an initial sink network *G*_*s*_. Next, we increase *r*_*s*_ by 0.2 m. Due to this increase, new nodes ν_*new*_ enter *r*_*s*_. Each ν_*new*_ is connected to any ν_*s*_ following preferential attachment. In a preferential attachment process, the higher the degree of node ν_*s*_ compared to the sum of all node degrees within *G*_*s*_, the more likely is ν_*new*_ to connect to ν_*s*_. After all ν_*new*_ were added to *G*_*s*_, *r*_*s*_ is increased again by 0.2 m. This process continues until *G*_*s*_ is of the same size as *CC*.
3. Repeat 2. for every *CC* in the swarm.

In Algorithm 1, *N*_*sink*_ is the size of the sink network *G*_*s*_, in terms of the number of nodes. Similarly, *N*_*CC*_ is the size of the selected connected component; *d*_*s*_ is the degree of node ν_*s*_, and $\underset{i}{\sum }d_{i}$ is the sum over all degrees in the sink-network. Note that the robot communication approaches the scale-free network topology only for large enough *CC*. However, due to the relatively small area of the nest the robots had a high tendency to self-aggregate into a giant connected component.

To test how successful Algorithm 1 was in generating a scale-free topology, we recorded the degree distributions at *t* = 10 of 1,000 simulation runs. At *t* = 10 the large majority of robots was still resting inside the nest, providing us with at least one large *CC*. Scale-free networks are characterized by the power law degree distribution. Thus, we tested whether our recorded degree distributions follow the power law using previously established statistical methods (Clauset et al., 2009; Broido and Clauset, 2019; Rausch et al., 2019a). Essentially, this statistical analysis is a highly rigorous power law fitting procedure that consists of three critical steps: (i) testing whether the shape of the distribution is due to random fluctuations, i.e., testing the *goodness-of-fit* given by a *p*-value. We proceed to the next step only if *p* < 0.1, otherwise the power law fit is considered unreliable. (ii) As the power law behavior is commonly found at the tail of the distribution, we proceed to the third step only if the data that is fit the power law behavior represents at least 10% of all data points. (iii) Finally, we compare the power law fit to other common distributions (such as the exponential or the log-normal) that may also tend to resemble a linear shape on a log-log scale (which is characteristic for the power law) (Clauset et al., 2009; Alstott et al., 2014). This is done by considering the log-likelihood ratio of each pair of distributions, which has a negative value if the distribution we compare the power law to is a significantly better fit. Consequently, the hypothesis that the data is power law distributed is not rejected only if this log-likelihood ratio is positive and only if we did not reject it at steps (i) and (ii). The result of the testing procedure can be captured by a numeric value to categorize whether the support for the hypothesis is not present, weak, moderate or strong (for more details see Rausch et al., 2019a). The test results for Algorithm 1 have shown a statistically sound support for the power law distribution in 76% of tests (we ran 1,000 tests), suggesting that Algorithm 1 was considerably successful in creating scale-free networks.

**Algorithm 1:**
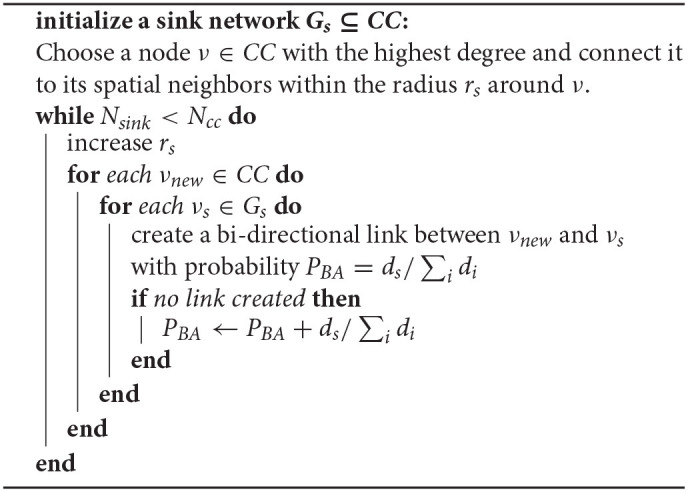
Pseudo-code for the implementation of the preferential attachment, executed at each time step.

Alternatively, one can use Algorithm 1 to construct networks with a degree distribution that is less skewed than power law and more symmetric around the mean degree, i.e., networks that resemble more closely the well-known small-world networks. To this end, one can simply replace the preferential attachment component $d_{s}/\underset{i}{\sum }d_{i}$ by a real number.

### 2.3. Swarm Behavior

At the swarm level, the foraging behavior emerges as a result of complex interactions between the robots as well as between robots and their environment. As mentioned above, we evaluate this performance in dynamic environments, in which the food density is subject to single and periodic changes. The quality of the emergent performance is evaluated with respect to the swarm response (adaptivity) to the changing number of items in the foraging environment. In particular, we define the swarm performance with respect to (i) the speed of the swarm's collective response, and (ii) the number of retrieved items. The collective response is quantified using the number of resting robots at any time step. For instance, in case of a sudden high availability of food items an ideal swarm's response would be to allocate more robots to the foraging state shortly after the increase in the number of food items is detected.

We borrow the term of settling time from control theory to measure the time of the swarm's collective response, referred to as the convergence time—i.e., the time the swarm needs to adapt the number of resting/foraging robots to any change in the items density. The settling time is defined as the time elapsed from the moment of applying a particular stimulus (i.e., changing the items' density) to the time the system output (i.e., number of robots *N*_*rest*_ that are in the resting state) reaches and remains within a specified margin of error. Hence, the time to convergence is computed as in the following:

(4) $$\begin{array}{l} \;t_{conv}=inf\left\{S\right\},\\ \text{where }S=\left\{t:|Fn\left(N_{rest}\left(t\right)\right)-Fn\left(N_{rest}\left(t_{steady}\right)\right)|<\zeta \right\}, \end{array}$$

where *inf*{*S*} is the greatest lower bound of the set *S*, and the set *S* includes all time steps *t* at which the difference between the transformed number of resting robots at a specific time step *N*_*rest*_(*t*) and the transformed number of resting robots at the steady state *N*_*rest*_(*t*_*steady*_) is smaller than a threshold ζ. In our study we set ζ = 0.1. Here, *t*_*steady*_ is the time step at which the system reaches its steady state. To compute the time to convergence, we use the matlab tool stepinfo^2^, that first applies *Fn*(...) to transform the input into a continuous representation. This transformation was used for *N*_*rest*_.

Finally, in addition to the convergence time, we investigate the swarm performance in terms of the number of retrieved items. The number of retrieved items is strongly related to the time to convergence, since a faster convergence implies a higher efficiency in retrieving items. We compute this performance measure using the cumulative sum of the items retrieved over time.

### 2.4. Simulation Setup

We ran the simulations using ARGoS^3^, a well-established physics-based simulator for swarm robotics (Pinciroli et al., 2012). The values of particular parameter settings that can be used to reproduce our simulations and results are listed in Table 1. Additionally, the reader is encouraged to find our project on the Open Science Framwork^4^ (Rausch et al., 2020) to download the development sources and run the simulations.

**Table 1:** Robot and arena parameters.

**Parameter**	**Value**
**ROBOT PARAMETERS**	
Physical avoidance range	0.1 m
Communication range	1.25 m
Maximum moving speed	1 m/s
Minimum resting time θ_*r*_	100 s
Minimum unsuccessful foraging time θ_*f*_	500 s
Minimum distancing time θ_*d*_	100 s
Individual cues *i*_*f*_,*i*_*r*_	0.01
Social cues *s*_*f*_,*s*_*r*_	{0.01, 0.25, 0.99}
**ARENA PARAMETERS**	
Total area of the arena *A*	50 × 50 m^2^
Area of the Nest *A*_*n*_	10 × 50 m^2^
Area of the Foraging environment *A*_*f*_	40 × 50 m^2^
Number of robots *N*_*robots*_	950
Number of items *N*_*items*_	30 or 300
Total experiment duration *T*	10^4^ ts

**Figure 2:**
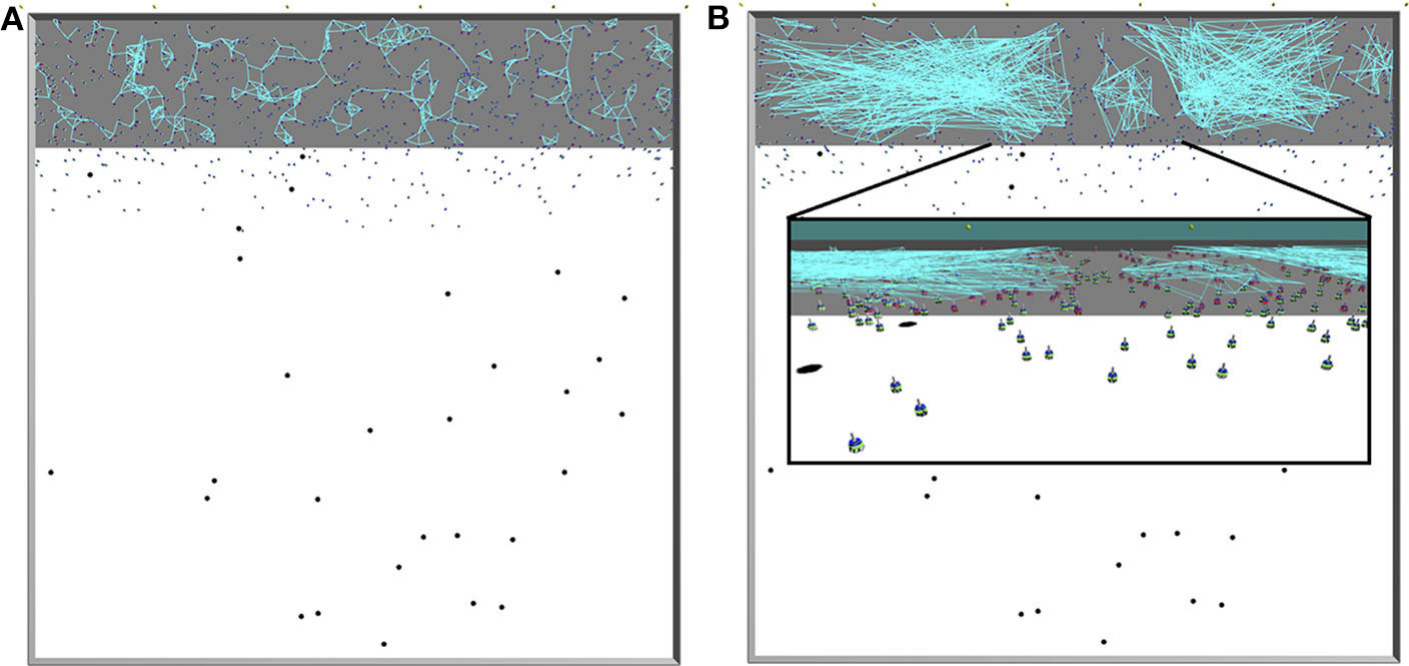
Illustrations of the arena taken from ARGoS simulations. Gray area: nest; white area: foraging environment; black dots: items; blue objects: Footbots; light-blue lines: communication (range-and-bearing) links. Top views onto the entire arena; the communication network is constructed in **(A)** using spatial network given by the local robot interactions, and in **(B)** using Algorithm 1; the inset shows a close-up view on the robots. In all figures, the communication links are formed *only* for *resting* robots *inside* the nest, as in our experiments moving robots neither broadcast nor listen to any messages.Therefore, it can happen that although a robot is within the communication range of another, no communication link is established between the two.

Figure 2 displays snapshots from simulations with proximity (Figure 2A) and scale-free (Figure 2B) networks. The square-shaped arena is of the size *L* × *L* (*L* = 50 m) and consists of the nest *A*_*n*_ = 10 × 50 m^2^ (gray colored floor in Figure 2) in addition to the foraging environment *A*_*f*_ = 40 × 50 m^2^ (white in Figure 2). Inside the foraging environment, food items are uniformly distributed. When a robot brings a food item to the nest, a new food item appears at a random location within the foraging environment, preventing item depletion that might lead the foraging activity to halt.

The robots are able to rapidly leave or return to the nest thanks to the phototaxis behavior. For that purpose, light beacons are installed on one side of the nest, opposite to the foraging environment (yellow dots at the top of Figure 2A or Figure 2B). Robots are repelled from the lights whenever they need to leave the nest, and attracted to the lights to return to the nest. The swarm consists of *N*_*robots*_ homogeneous robots (we use Footbots; Bonani et al., 2010). Robots are equipped with probabilistic controllers, which tune their behavior to forage or rest based on the above mentioned probabilities (i.e., *P*_*r*→*f*_ and *P*_*f*→*r*_).

To implement the proposed networks (i.e., scale-free and proximity), we utilize the range-and-bearing medium (that includes sensor and actuator) provided in ARGoS. However, this communication medium is used differently for the two networks. In the case of proximity networks, the communication range of the range-and-bearing medium is set to 1.25 m (as we can see in Table 1). In the case of the scale-free networks, at each time step, we first obtain the connected components using the spatial proximity network, where the robots communicate via the range-and-bearing medium within a radius of 1.25 m. In the same time step, for each of these connected components, we create a scale-free network in which the connections can span over the entire length of the nest, if the connected component spans over that area. Thus, the resulting scale-free networks can include much longer ranges than 1.25 m. For implementing such a communication topology in real-world swarms, it is possible to apply other communication systems than the range-and-bearing medium, such as other radio communication technologies (e.g., the well-established wifi Li et al., 2008), shared memory (Bayındır, 2016) or promising concepts such as the augmented reality for Kilobots (ARK) (Reina et al., 2017).

## 3. Results and Discussion

The goal of this study is to investigate the influence of the scale-free topology on the collective performance and response of a swarm foraging in a dynamic environment. The dynamics of the environment is modeled in terms of single and periodic changes in the food density. In robot swarms, the interaction among individuals is mostly modeled using local communications, where each robot has a limited communication range. The communication range is usually much smaller than the dimension of the world. The robot's neighborhood is defined as the set (or a subset) of robots that is located within its communication range. In this study, besides local interactions, we make use of the well-known preferential attachment mechanism (applied in Algorithm 1, see section 2.2) to construct a scale-free topology that accelerates information sharing. Hence, we investigate whether it may improve the efficiency of the swarm collective response to environmental dynamics.

As mentioned above, we define the collective response in terms of the number of resting robots and measure it as the change in this number over time. In our experiments, initially, the entire swarm is in the resting state. In the following, a transient period begins, during which the swarm displays oscillations at the group level. First, almost all robots begin foraging during the first 500 time steps (ts)—Note that a simulated time step is one second, with one tick per second. Within the subsequent ≈ 500 ts most of the swarm individuals come back to the nest and switch to resting. Even though such collective behavior oscillates over several following time periods—due to the probabilistic nature of the robot controller—the coherence increases rapidly and the swarm converges on a relatively stable number of resting robots. The duration of this transient period is mostly shorter than 5·10^3^ ts, after which we begin our measurements. Finally, based on preliminary results, we set the swarm size to *N* = 950, which balances physical interference with swarm performance and delivers a sufficiently large number of samples for statistically sound analysis.

We use two experimental settings. In the first setting, after the system converges on a number of resting robots *N*_*rest*_ (number of foraging robots is then *N*_*forg*_ = *N* − *N*_*rest*_), a single external stimulus is applied. This stimulus represents an increase in the number of food items *N*_*items*_ by the factor of 10 (from 30 to 300 items) at a particular time point *t*_*crit*_. In the second experimental settings, we challenge the swarm further by applying a periodic change in the density of the food items, hence the benefit of a quicker response becomes clearer. The periodic change is applied over periods of 2500 ts and can be of two types, either increasing or decreasing the number of food items *N*_*items*_, always by a factor of 10.

In each of the two experimental settings, two interaction networks are implemented, proximity network (emerging from local interactions), and scale-free network (generated using preferential attachment). As mentioned above, for the construction of scale-free networks, the connected components of the robots resting at the nest site are used to impose the network topology. Over these networks the robots exchange specific information about their success or failure of the latest foraging attempt seeking an accurate estimation of the current situation in the foraging environment.

According to our experiments, there are two main cases, in which the influence of the communication topology is negligible. These are (i) small social cues (i.e., with *s*_*f*_ and *s*_*i*_ values smaller than 0.01), and (ii) small number of resting robots *N*_*rest*_. The first case is straightforward, as the social cues decrease, the impact of the information obtained from other robots decreases, and hence the impact of the interaction network on the emergent dynamics vanishes. The second case is associated with the particular implementation of the scale-free communication network in the nest. Since the construction of this network relies on the connected components present in the nest at every time step, small numbers of resting robots result in scaling down the size of such connected components and hence topological contribution becomes negligible. Therefore, as we aim to investigate the influence of the interaction network on the emerging dynamics, we consider those cue configurations in which the social feedback of the robot's neighborhood has a distinguishable role in shaping its decision. This is achieved by setting the social cues to have a clear advantage over the individual cues—i.e., *s*_*f*_ ≫ *i*_*f*_, *s*_*r*_ ≫ *i*_*r*_. For an extensive discussion on the impact of cue values on swarm behavior in a similar settings of the foraging task the interested reader is referred to Liu et al. (2007) and Rausch et al. (2019a). For the reasons mentioned above, we set the cue values to *s*_*f*_ = 0.25, *s*_*r*_ = 0.25, *i*_*f*_ = 0.01, *i*_*r*_ = 0.01. Nevertheless, further below we will additionally compare our results to those obtained with more extreme values of the social cues, i.e., *s*_*f*_ = 0.01, *s*_*r*_ = 0.01 and *s*_*f*_ = 0.99, *s*_*r*_ = 0.99.

**Figure 3:**
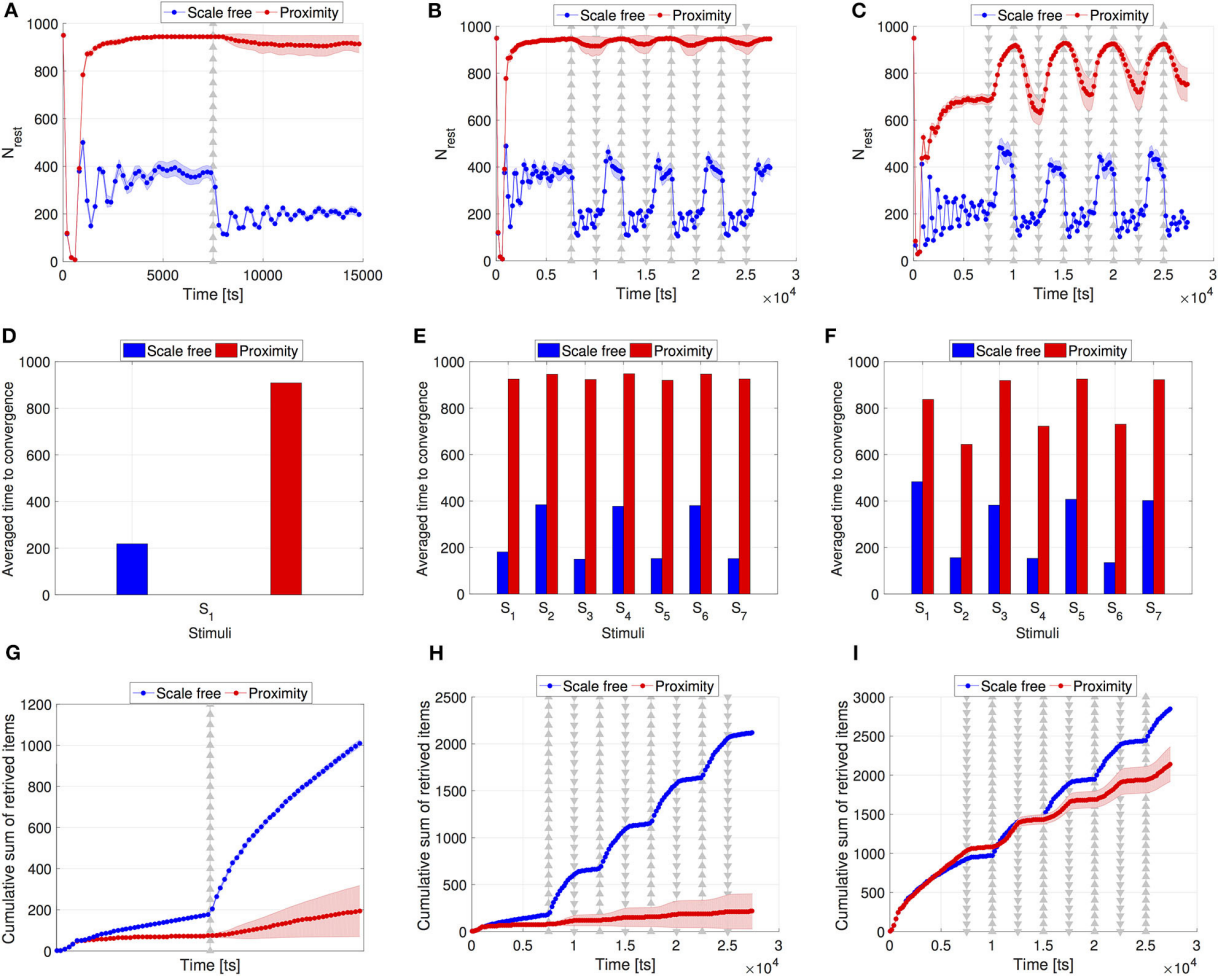
Swarm performance comparison between the scale-free networks (blue) and the proximity networks (red). (Top) Swarm collective response in terms of *N*_*rest*_. **(A)** Single stimulus of item gain from *N*_*items*_ = 30 to *N*_*items*_ = 300 at *t*_*crit*_ = 7, 500 ts, and **(B)** multiple stimuli are executed in intervals of Δ*t*_*crit*_ = 2, 500 ts. The items are repeatedly increased to *N*_*items*_ = 300 (indicated by ▵) or reduced to *N*_*items*_ = 30 (indicated by ▿). **(C)** Similar setting to **(B)**, but starting from *N*_*items*_ = 300 and changing the items in an inverse order, as indicated by the ▵ and ▿ markers. (Center) Swarm convergence time. **(D)** Single stimulus of item gain, *S*_1_ is the index for the stimulus applied at *t*_*crit*_ = 7, 500. **(E)** Multiple stimuli where items are repeatedly increased or reduced. *S*_1…7_ correspond to the seven stimuli applied between *t*_*crit*_ = 7500 ts and *t* = 25, 000 ts in intervals of Δ*t*_*crit*_ = 2, 500 ts, as in **(B)**. **(F)** Similar to **(E)** but with an inverse order, as in **(C)**. (Bottom) Cumulative sum of the retrieved items. **(G)** Scenario with a single stimulus. **(H)** Scenario that starts with *N*_*items*_ = 30, as in **(B)**. **(I)** Scenario that starts with *N*_*items*_ = 300, as in **(C)**. In **(A–C)** and in **(G–I)**, shaded areas indicate the confidence interval of 95%. All results were averaged over 30 runs.

The plots in Figure 3 depict results obtained over 30 runs. They compare the emergent collective response of the swarm to a single stimulus (i.e., change in food density) as well as to multiple stimuli when individuals interact locally in comparison to interacting via scale-free topologies. Firstly, our results reveal a clear impact of the network structure on the robot activation level across all types of stimuli (i.e., increasing or decreasing food item density). This is illustrated through the number of resting robots being considerably smaller when using the scale-free network as opposed to the proximity network throughout the entire simulation time (see Figures 3A,B). Proximity networks in Figure 3B show a non-adaptive swarm behavior that is largely due to the very low number of foraging robots. When there are too few foraging robots, the system tends to approach a global absorbing state in which robots cease to switch to foraging. In case of proximity networks in Figure 3B, this tendency toward the global resting state is due to the initial low density in food items (i.e., *N*_*items*_ = 30). Low *N*_*items*_ leads to a large number of failed attempts to find and retrieve them. Consequently, this increases *P*_*f*→*r*_ up to its maximum *P*_*f*→*r*_ = 1, pushing the robots to keep resting. Thus, the subsequent increase in items to *N*_*items*_ = 300 is not sensed by the swarm. As an example, this behavior is evident at *t* = 7, 500 ts when *N*_*rest*_ did not decrease in response to the increasing *N*_*items*_.

Therefore, it is important to consider the robustness of the swarm behavior to initial conditions, prior to the external stimulus. To this end, we inverted the changes of *N*_*items*_, starting with *N*_*items*_ = 300, reducing it to *N*_*items*_ = 30 at *t* = 7, 500 ts, then increasing it back to *N*_*items*_ = 300, etc… Under this specific setting, foraging robots have a higher likelihood to find items than when the initial item density is as low as *N*_*items*_ = 30. Consequently, the returning robots broadcast a larger number of “success” messages, increasing the robots' probability to switch to foraging (*P*_*r*→*f*_). Figure 3C shows that this configuration of the initial conditions led to an adaptive swarm behavior for the case of proximity networks. This adaptive behavior comes with a reduced time to convergence (see Figure 3F vs. Figure 3E) and a significantly higher number of retrieved items (see Figure 3I vs. Figure 3H). Nevertheless, with scale-free networks the collective response not only remained more rapid but also appeared to be more robust to the initial conditions of the system, as the trajectory of *N*_*rest*_ in Figure 3C is qualitatively similar to Figure 3B. Nevertheless, the scale-free networks display higher fluctuations of *N*_*rest*_ compared to the relatively coherent decision achieved when using proximity networks (Figures 3A–C). This is due to the high impact that a single hub can have on a large population of the swarm.

The key contribution of the network topology is reflected in the time the swarm requires to build up its collective response. When using scale-free networks, hubs—i.e., robots with an exceptionally high connectivity degree—help accelerate the information propagation in two manners: (i) due to their high connectivity degree, their individual experience is shared with a large number of robots within one time step. (ii) Their presence creates a shorter average path of the network compared to proifbximity networks, which allows any two robots to exchange information over a smaller number of hops (i.e., within fewer time steps). As mentioned above, we use the settling time defined in Equation (4) to compute the swarm's convergence time after each stimuli—i.e., change in the items' density. Figure 3D shows the time it took the swarm to converge to a steady number of resting/foraging robots after increasing the items at the foraging area from *N*_*items*_ = 30 to *N*_*items*_ = 300 at time step Δ*t*_*crit*_ = 7, 500. Figures 3E,F show the same measure for the repeated stimuli of items increase and decrease, starting from *N*_*items*_ = 30 (Figure 3E) and *N*_*items*_ = 300 (Figure 3F). In all three findings, Figures 3D–F, we can notice the significantly shorter convergence time when robots in the nest are communicating using the scale-free network in comparison to the proximity network. These results suggest a higher level of swarm adaptivity to dynamic environments under scale-free communications. Furthermore, as shown in Figures 3G–I, using scale-free networks the cumulative sum of the retrieved items is either considerably higher from the beginning or at the later stages of the experiment, compared to the scenarios with proximity networks.

An important aspect to notice is the physical division between the site at which the information is to harvest (i.e., the foraging environment), and the site at which the information is to exchange (i.e., the nest). Usually, the communication speed is considerably higher than motion speed. However, specifically in the foraging scenario, the communication speed is limited by the motion speed, since it is necessary for the robot to travel across the foraging environment to reach the nest, where it can start communicating. One of the clear consequences of this important remark is that even for the case of scale-free networks where the collective response is accelerated, there is a considerably faster swarm reaction to an increase in the food density compared to the reaction to a decrease (see the blue line in Figure 3B). Before the increase of food items, there were few foraging robots. Those robots consumed time to return to the nest, switch to resting, inform their neighbors about their foraging experience, and, ultimately, convince more robots to leave the nest in case of a successful foraging attempt. For scale-free networks this resulted in a rapid activation of resting robots. Differently, collective reaction slowed down when the environmental change was a decrease in food items. This behavior can be explained as follows: the large number of robots foraging while the food density was high experienced the drop in the food density through their failed foraging attempts. Upon returning to the nest, these robots led to considerably higher crowding at the nest entrance. This prolonged the time that the robots needed to enter the nest and start communicating. Moreover, the higher *N*_*rest*_ the higher the likelihood that there is one, giant, connected component inside the nest, spanning over a large number of robots. If such a network is scale-free, the hubs have a high chance of influencing many robots to switch to foraging. By contrast, a low *N*_*rest*_ often led to fragmented networks, reducing the influence of hubs, lowering the number of switching robots and, thus, slowing down the collective response compared to a high *N*_*rest*_. Hence, the collective response time—even when using scale-free networks—is longer when there are many robots foraging.

**Figure 4:**
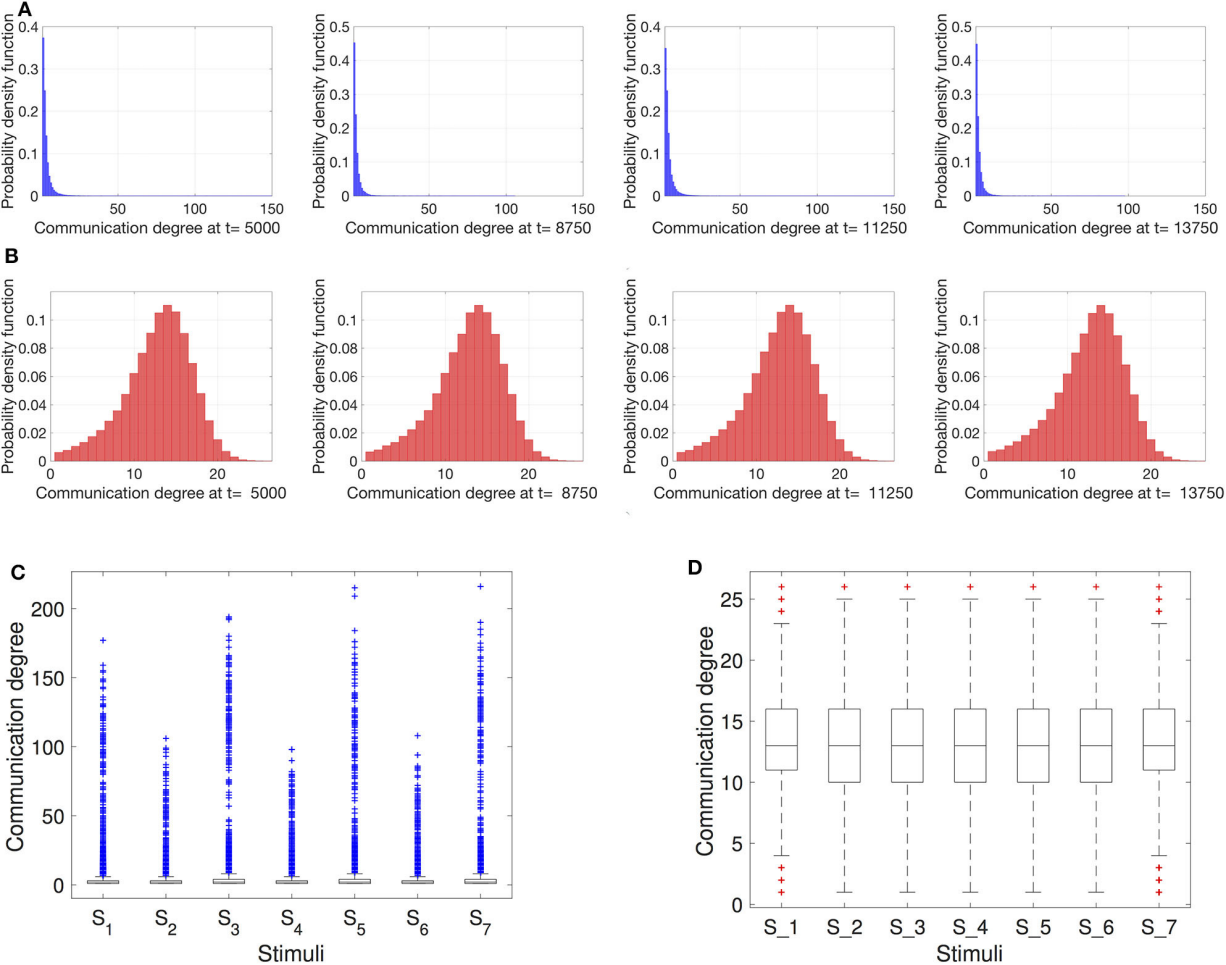
Degree distributions of the networks within the nest at different time instances. **(A)** Scale-free networks; **(B)** Proximity networks. At *t* = 5, 000 ts and *t* = 11, 250 ts there are *N*_*items*_ = 30 to retrieve, while at *t* = 8, 750 ts and *t* = 13, 750 ts the item count is *N*_*items*_ = 300. Additionally, box plots for the **(C)** scale-free and **(D)** proximity networks illustrate the presence of outliers for the different onsets of stimuli *S*_1…7_ (starting at *t*_*crit*_ = 7, 500 ts and occurring in intervals of Δ*t*_*crit*_ = 2, 500 ts). As expected, in contrast to the proximity networks, in case of scale-free networks, the outliers (indicated by the + markers) are so extreme that the boxes containing the mean values are barely recognizable at the bottom of plot **(C)**.

To obtain a closer look at the interaction network topology, we can analyze the degree distributions of the resting robots interacting inside nest. We draw the degree distributions for different time steps that are selected when the item density was both high (i.e., 300 items) and low (i.e., 30 items). As we can see in Figure 4A, scale-free networks strongly resemble a power-law distributed degree for all time steps at which the networks are recorded. Similar consistent is the degree distribution of the proximity networks in Figure 4B for all tested time steps. However, the degree distribution here appears closer to a Gaussian distribution which is more symmetrical around the mean than the scale-free network and has fewer outliers. To get a clearer look at the outliers, in Figures 4C,D, we show the communication degree using boxplots. For the scale-free networks the density of outliers is notably large, the most extreme among those are the hubs in the network. We can also notice a clear trend of a higher number of hubs when the number of resting robots *N*_*rest*_ is higher due to low *N*_*items*_. This density of outliers changes periodically between the external stimuli *S*_*i*_ together with *N*_*rest*_. In the case of proximity networks, the boxplots show a relatively low density of outliers and negligible changes with *S*_*i*_.

Additionally, it is worthwhile considering the effect of rewiring on the collective response. As elaborated in section 2.2, Algorithm 1 is applied at every time step as the robots are in motion. However, because Algorithm 1 has a stochastic component, the resulting network at time step *t* is very likely to be different from *t* − 1. Such dynamic rewiring increases the probability that two remote robots share a link. Consequently, a random robot is more likely to obtain information from spatially uncorrelated sources, i.e., it obtains a sample that is more representative of the swarm opinion. This resembles the common “random mixing” paradigm often found in swarm robotics, stating that an encounter probability between two robots is the same for any pair of robots. Thus, the adaptive behavior that follows from using Algorithm 1 could be largely attributed to this rewiring-induced opinion mixing.

**Figure 5:**
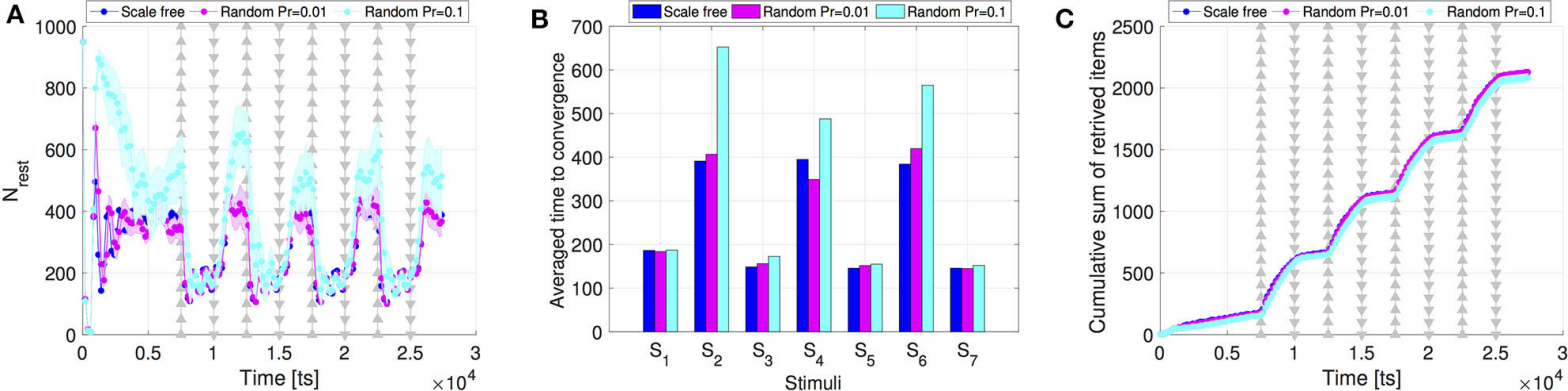
Comparison of the **(A)** swarm collective response, **(B)** time to convergence, and **(C)** swarm performance, between scale-free networks and random networks created with ρ = 0.01 and ρ = 0.1.

To examine whether this may indeed be the case, we ran simulations with a modified version of Algorithm 1 where we replaced the preferential attachment component $d_{s}/\underset{i}{\sum }d_{i}$ by a real number ρ ∈ {0.01, 0.1}. Note that while this modification aims at altering the network topology, the resulting alternative networks are still regenerated at each time step, similar to scale-free networks, i.e., the notion of rewiring is preserved. The results are shown in Figure 5. The similarity to the scale-free networks scenario is particularly striking for ρ = 0.01. When *N*_*rest*_ is low, it becomes difficult to separate a scale-free network (where the degrees are power law distributed) from a small-world network (where the degree distribution is much less skewed, i.e., more symmetric around the mean value). Therefore, for low *N*_*rest*_ the impact of the preferential attachment component in Algorithm 1 can be well-approximated by a constant such as ρ = 0.01. More importantly, it shows that the strong effect that dynamic rewiring has on swarm adaptivity and collective response.

**Figure 6:**
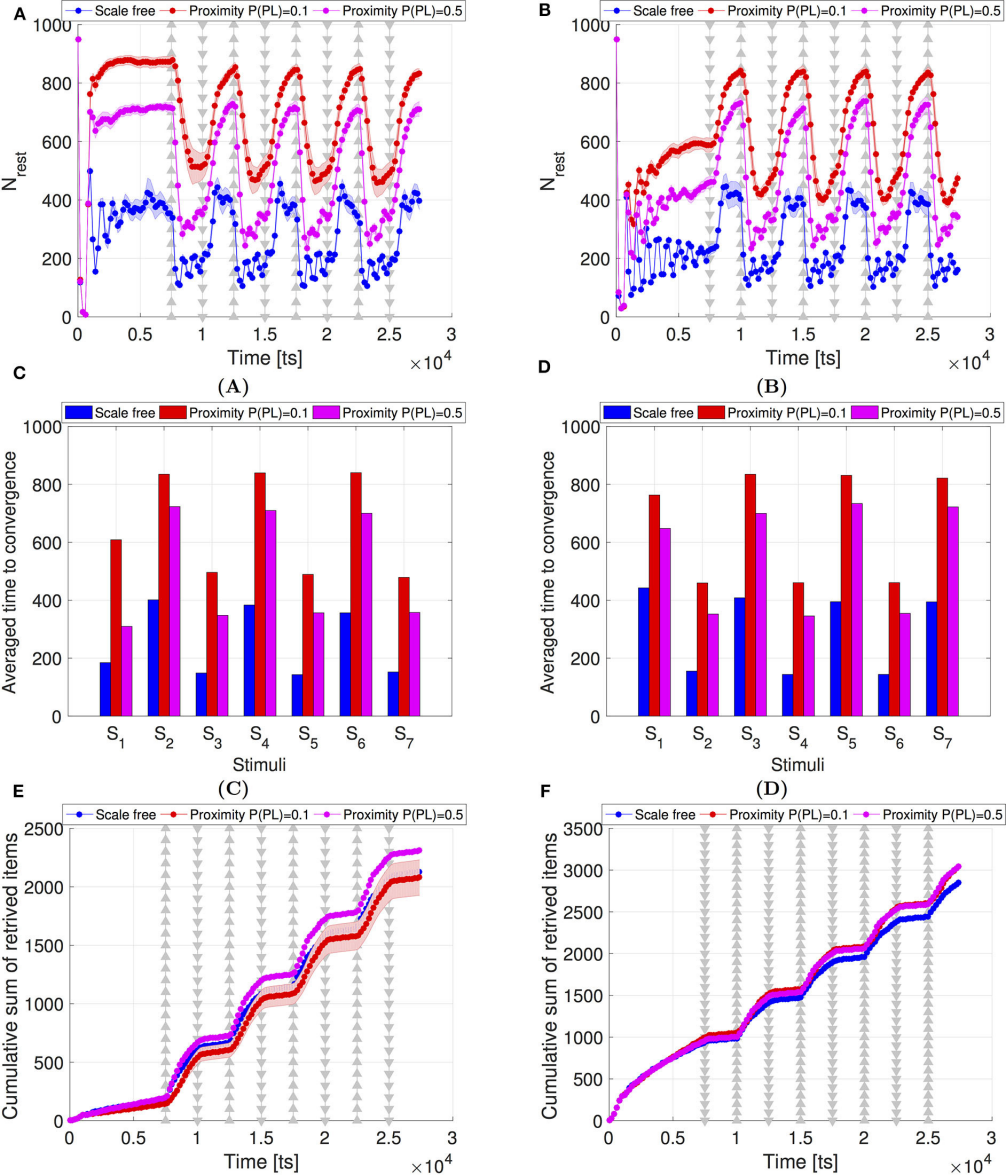
Swarm performance comparison between the scale-free networks (blue) and the proximity networks in presence of packet loss, with packet loss probability *p*_*pl*_ = 0.1 (red) and *p*_*pl*_ = 0.5 (magenta). The number of items is repeatedly increased to *N*_*items*_ = 300 (indicated by ▵) or reduced to *N*_*items*_ = 30 (indicated by ▿). These repeating changes occur in intervals of Δ*t*_*crit*_ = 2, 500 ts, starting at *t*_*crit*_ = 7, 500 ts. (Left) Scenario with initially *N*_*items*_ = 30. (Right) Scenario with initially *N*_*items*_ = 300; **(A,B)** Swarm collective response in terms of *N*_*rest*_. **(C,D)** Swarm convergence time. *S*_1…7_ correspond to the seven stimuli between *t*_*crit*_ = 7, 500 ts and *t* = 25, 000 ts. **(E,F)** Cumulative sum of the retrieved items. In **(A,B,E,F)**, the shaded areas indicate the confidence interval of 95%. All results represent averages over 30 runs.

A feature that frequently occurs in realistic communication is the packet loss. It occurs when a robot fails to receive a message broadcast by a neighbor, due to radio-frequency interference or due to overflow of a robot's receiver queue. We implemented packet loss events by allowing the robots to ignore incoming messages with probability *p*_*pl*_. Figure 6 shows the results for the proximity and scale-free networks with *p*_*pl*_ ∈ {0.1, 0.5}. Surprisingly, the swarm adaptivity considerably improves in case of proximity networks, while with scale-free networks the swarm remains more robust to the influence of the packet loss. Higher probabilities of packet loss appears to shorten the time to convergence and slightly increase the number of collected items. One possible explanation for this behavior could be that by probabilistically ignoring incoming messages the robots become to some extent able to reduce the correlation between their behavior and that of their spatial neighbors. Synthetically generated networks, such as the scale-free networks considered in this study, represent an extreme case of such spatial decorrelation. In contrast, in proximity networks and absence of packet loss, spatial correlations are very high, leading to feedback mechanisms that reduce sensitivity to new information. The presence of packet loss appears to create a middle ground that bolsters the adaptive behavior at the swarm level. However, we only tested two values of *p*_*pl*_ and it is possible that for *p*_*pl*_ > 0.5 inverse effects could be observed. Finally, when resting state can be associated with low energy consumption, the behavior of the system in the presence of here considered *p*_*pl*_ may demonstrate a high level of efficiency, in terms of increasing *N*_*rest*_ while preserving the high number of retrieved items. Nevertheless, as mentioned above, the detailed investigation of the influence of packet loss is beyond the scope of the current study and future research is needed to confirm the generality of our findings^5^. Moreover, here we consider constant values of *p*_*pl*_ that are the same for every robot in the swarm and that do not change based on the location of the robot or the number of communication links. In contrast, in more realistic settings not only the packet loss but also *p*_*pl*_ itself may have fluctuating values depending on the situation and both could be profoundly difficult to control.

**Figure 7:**
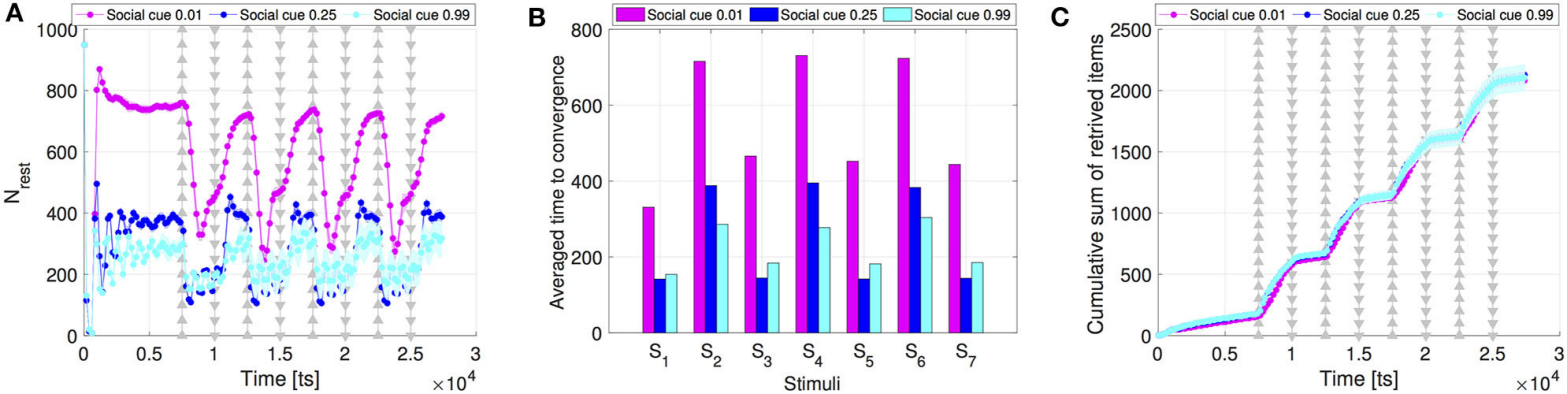
Comparison of the **(A)** swarm collective response, **(B)** time to convergence, and **(C)** swarm performance, between different values of social cues for swarms communicating through scale-free networks. Apart from *s*_*f*_ = *s*_*r*_ = 0.25 we consider two extreme cases: low values (*s*_*f*_ = *s*_*r*_ = 0.01) and high values of social cues (*s*_*f*_ = *s*_*r*_ = 0.99). All results were averaged over 30 runs.

Finally, we compare the intensity of the collective response resulting from different social cues. As mentioned above, social cues are the main driver of the dynamics to build up a faster response over the interaction network. Our results show that higher social cues lead to a higher activation of the resting robots, see Figure 7 that shows the activation of the resting robots when setting *s*_*f*_ = 0.99, *s*_*r*_ = 0.99 in comparison to the setting *s*_*f*_ = 0.01, *s*_*r*_ = 0.01 (results are averaged over 30 runs). High social cues activate considerably more resting robots (i.e., reduces number of resting robots) than low cue values (Figure 7A). However, the convergence time with high cue values is comparable to the previously considered default case of *s*_*f*_ = *s*_*r*_ = 0.25 (see Figure 7B). The number of collected items overlaps for all three cue values (see Figure 7C).

## 4. Conclusion

The goal of this study is to investigate the role of network topology in influencing the propagation of information in a foraging scenario with changing the availability of food items. Therefore, we have addressed scenarios with dynamic environments, a realistic aspect of most real-world applications. We considered two types of changes: a single abrupt change (referred to a single stimulus) and periodic changes (multiple stimuli). We aimed to examine how scale-free networks, in particular, may accelerate the spreading of information and hence enable a quicker collective response than proximity networks to the global changes.

We have implemented scale-free networks across the robots resting in the nest, as the nest is usually the part of the environment in which communication takes place. We applied the well-known preferential attachment technique to construct the scale-free topology. Following preferential attachment, the probability of connecting to a robot is proportional to its current connectivity degree. Therefore, a number of robots emerge to have a relatively high degree of connectivity, those are referred to as the hub robots. When the density of food items changes at the foraging environment, and this change is reflected in the robots' experience, scale-free networks enable a faster spreading of this information in the nest. This led to a faster collective response compared to the scenarios in which interactions between the resting robots were implemented using proximity networks.

Our results suggest that the use of scale-free networks can improve the collective response of the swarm to changes in their dynamic environment, by improving the spread of shared information and reducing the spatial correlation in the robots' decisions. These two desired features in collective systems are achieved due to the introduced possibility to communicate over long distances, as well as due to the dynamic rewiring of the interaction network at every time step as a consequence of robot motion. These insights were obtained by comparing the swarm behavior in scenarios with and without systematic packet loss, in addition to comparing the swarm performance between scenarios with scale-free networks and with alternative random networks. Furthermore, our findings showcase the effect of social cues on the intensity of the collective response in presence of scale-free networks. Our results show that higher social cues lead to a higher activation of the resting robots, due to the increased influence of their neighbors' experience.

Although scale-free networks have shown to equip the swarm with a quicker reaction to changes in dynamic environments—studied for the collective foraging task—this came at the cost of the coherence of the collective response. Scale-free topologies led to more fluctuations of the swarm decision (whether to rest or to forage). These fluctuations can be explained in terms of the high influence of particular individuals (i.e., the hubs) on the opinions of a large population of the resting robots. Two particularly promising research directions for future work include the design of self-organized algorithms to implement scale-free topologies in robots swarms. Additionally, the design of efficient individual decision mechanisms that helps the collective response to demonstrate a higher stability. Finally, generalizing this study to other collective tasks such as site selection, flocking, and others may also lead to new interesting insights.

## Data Availability Statement

The original contributions presented in the study are publicly available. This data can be found here: https://osf.io/48b9h/.

## Author Contributions

All authors contributed conception and design of the study. PS provided funding and administered the project. YK and PS supervised the study. IR implemented the simulation setup, performed the simulations, and the formal data analysis. YK wrote the first draft of the manuscript. YK and IR continuously discussed and elaborated on the design parameters and obtained results, which led in some cases to further implementations and analysis. YK and IR wrote most sections of the manuscript and analyzed the experimental data. IR and YK critically reviewed and edited the manuscript. All authors approved the submitted version.

## Conflict of Interest

The authors declare that the research was conducted in the absence of any commercial or financial relationships that could be construed as a potential conflict of interest.

## Footnotes

^1^https://osf.io/48b9h/

^2^https://www.mathworks.com/help/control/ref/stepinfo.html

^3^http://www.argos-sim.info/

^4^https://osf.io/48b9h/

^5^To this end, the interested reader is encouraged to use our publicly available resources provided on https://osf.io/48b9h/.
